# A Novel Polyester Hydrolase From the Marine Bacterium *Pseudomonas aestusnigri –* Structural and Functional Insights

**DOI:** 10.3389/fmicb.2020.00114

**Published:** 2020-02-13

**Authors:** Alexander Bollinger, Stephan Thies, Esther Knieps-Grünhagen, Christoph Gertzen, Stefanie Kobus, Astrid Höppner, Manuel Ferrer, Holger Gohlke, Sander H. J. Smits, Karl-Erich Jaeger

**Affiliations:** ^1^Institute of Molecular Enzyme Technology, Heinrich Heine University Düsseldorf, Jülich, Germany; ^2^Center for Structural Studies, Heinrich Heine University Düsseldorf, Düsseldorf, Germany; ^3^Institute for Pharmaceutical and Medicinal Chemistry, Heinrich Heine University Düsseldorf, Düsseldorf, Germany; ^4^Institute of Catalysis, Consejo Superior de Investigaciones Científicas, Madrid, Spain; ^5^Institute of Biological Information Processing (IBI-7: Structural Biochemistry), John von Neumann Institute for Computing and Jülich Supercomputing Centre, Forschungszentrum Jülich GmbH, Jülich, Germany; ^6^Institute of Biochemistry, Heinrich Heine University Düsseldorf, Düsseldorf, Germany; ^7^Institute of Bio- and Geosciences IBG-1: Biotechnology, Forschungszentrum Jülich GmbH, Jülich, Germany

**Keywords:** *Pseudomonas aestusnigri*, marine bacteria, polyester degradation, polyethylene terephthalate, PET, crystal structure

## Abstract

Biodegradation of synthetic polymers, in particular polyethylene terephthalate (PET), is of great importance, since environmental pollution with PET and other plastics has become a severe global problem. Here, we report on the polyester degrading ability of a novel carboxylic ester hydrolase identified in the genome of the marine hydrocarbonoclastic bacterium *Pseudomonas aestusnigri* VGXO14^*T*^. The enzyme, designated PE-H, belongs to the type IIa family of PET hydrolytic enzymes as indicated by amino acid sequence homology. It was produced in *Escherichia coli*, purified and its crystal structure was solved at 1.09 Å resolution representing the first structure of a type IIa PET hydrolytic enzyme. The structure shows a typical α/β-hydrolase fold and high structural homology to known polyester hydrolases. PET hydrolysis was detected at 30°C with amorphous PET film (PETa), but not with PET film from a commercial PET bottle (PETb). A rational mutagenesis study to improve the PET degrading potential of PE-H yielded variant PE-H (Y250S) which showed improved activity, ultimately also allowing the hydrolysis of PETb. The crystal structure of this variant solved at 1.35 Å resolution allowed to rationalize the improvement of enzymatic activity. A PET oligomer binding model was proposed by molecular docking computations. Our results indicate a significant potential of the marine bacterium *P. aestusnigri* for PET degradation.

## Introduction

The modern society depends on the production and use of synthetic polymers which are uniformly present in both, basic and high-tech applications. The low production costs for plastic made from fossil feedstock and the high durability of the material are major advantages but have become a burden for the global ecosystem. Plastic waste is produced at a much faster rate than it is recycled (Moharir and Kumar, 2019); hence, it is disposed in landfills at a large extend where it can take centuries to degrade completly. Morever, small plastic particles, so-called microplastics, usually evade municipal waste collection, being directly released into waste water and spread easily around the globe (Rochman, 2018). Hence, plastic waste accumulates in the environment to a large extent with very slow biodegradation to occur (Lebreton et al., 2018).

Where most plastics are inert polyolefins, consisting of carbon-carbon bonds, heteroatomic plastics like polyamides, polyurethanes and polyesters provide chemical groups of higher reactivity and thus are more easily degraded biologically (Wei and Zimmermann, 2017). The most abundant polyester plastic, present for example in packaging waste, is polyethylene terephthalate (PET) (Adrados et al., 2012). In the European Union, five million metric tons of this polyester were used for the production of plastics in 2017 (PlasticsEurope, 2018).

Enzymes catalyzing the degradation of polyesters such as polycaprolactone (PCL), polylactic acid (PLA), or PET are found within the class of carboxylic ester hydrolases (E.C. 3.1.1), most of them are classified as cutinases (E.C. 3.1.1.74), enzymes naturally adapted to act on polymeric ester substrates, e.g., the wax cuticle of plants (Nikolaivits et al., 2018). Studies on the identification of polyester degrading enzymes have shown that only a small fraction of carboxylic ester hydrolases is able to degrade synthetic polyester substrates. In a comprehensive metagenomics screening study, a subset of 23 carboxylesterases were tested for PLA hydrolysis, yielding seven positive hits (Popovic et al., 2017). More recently, by screening of over 200 different purified hydrolases for activity on synthetic polyesters, 36 positive enzymes were identified of which 10 enzymes showed high activity on multiple polyester substrates (Hajighasemi et al., 2018). For PET degradation, comprehensive activity-based screening studies are missing. However, a bioinformatics study using a hidden Markov model succeeded to identify PET hydrolase genes using the UniProtKB database and more than 100 metagenome datasets, many of which originated from marine sources (Danso et al., 2018). The reported frequency of PET hydrolases was, dependent on the origin of the metagenomic sample, between 0.0001 and 1.5 hits per megabases of sequence, with the highest hit rate in a metagenome obtained from an oil polluted environment (Danso et al., 2018). In contrast to the marine origin of many predicted PET hydrolases, most of the PET degrading enzymes studied so far originate from terrestrial sources, with Cut190 from *Saccharomonospora viridis* (Kawai et al., 2014), Tha_Cut1 from *Thermobifida alba* (Ribitsch et al., 2012), Thc_Cut1 and Thc_Cut2 from *T. cellulosilytica* (Ribitsch et al., 2012), Tfu_0883, Tfu_0882 and TfCut2 from *T. fusca* (Chen et al., 2010; Roth et al., 2014) and LCC identified from a leaf-branch compost metagenome (Sulaiman et al., 2012) for example. All these enzymes share a characteristic thermostability which is in line with the lifestyle of their thermophilic host organism or the respective environment. This feature is beneficial for the degradation of solid PET, since the glass transition temperature of PET, i.e., the temperature where the polymer becomes flexible and thus more accessible to enzymatic degradation, is about 75°C (Wei et al., 2019a). However, biodegradation of PET can also occur at lower temperatures, as demonstrated with PETase from *Ideonella sakaiensis*, the first such enzyme originating from a mesophilic organism (Yoshida et al., 2016). This enzyme outcompetes other cutinases for the hydrolysis of crystalline PET at 30°C as demonstrated in a comparative study with the leaf-branch compost cutinase (LCC) and a cutinase from the *Thermobifida* group (Yoshida et al., 2016). The elucidation of PETase three-dimensional structures by different groups (Han et al., 2017; Austin et al., 2018; Chen et al., 2018; Joo et al., 2018; Liu B. et al., 2018; Liu C. et al., 2018; Palm et al., 2019), lead to a proposal for the degradation mechanism and structural hallmarks responsible for superior activity as reviewed by Taniguchi et al. (2019). Structural features compared to other cutinase structures include an additional disulfide bond for improved stability at the position of the active site histidine, allowing for increased flexibility of the adjacent extended loop region (Fecker et al., 2018), thus facilitating the interaction with the polymer (Joo et al., 2018). Based on sequence and structural information, Joo et al. defined different types of PET degrading enzymes: Most known cutinases were assigned to type I, and enzymes possessing an additional disulfide bond and an extended loop region were assigned to type II, which was subdivided into types IIa and IIb based on the amino acid composition of respective regions (Joo et al., 2018). Crystal structures are published for several representatives of type I (Roth et al., 2014; Sulaiman et al., 2014; Miyakawa et al., 2015; Ribitsch et al., 2017), for type IIb just one enzyme with solved crystal structures exists (Han et al., 2017; Austin et al., 2018; Fecker et al., 2018; Joo et al., 2018; Liu B. et al., 2018; Liu C. et al., 2018; Palm et al., 2019), and, to the best of our knowledge, no crystal structure is known for a type IIa enzyme.

Recently, we observed that the marine bacterium *Pseudomonas aestusnigri* showed polyester degrading activity (Molitor et al., 2020). In this study, we identified the polyester hydrolase named PE-H which belongs to type IIa of PET hydrolases and demonstrated its activity toward PET as a substrate. We also report on the first crystal structure of a type IIa PET hydrolase. By a site-directed mutagenesis approach, inspired by known PETase structural features, we obtained a PE-H variant with significantly improved activity. The crystal structure of this variant was solved as well allowing us to rationalize our biochemical findings.

## Materials and Methods

### Enzyme Production and Purification

#### Construction of the Expression Plasmid

The gene coding for the enzyme PE-H (locus tag B7O88_RS11490 of NCBI Reference Sequence NZ_NBYK01000007.1) was cloned into expression vector pET-22b(+) (Novagen) in frame with the vector-encoded hexa histidine tag utilizing *Xba*I and *Xho*I endonuclease restriction sites (Green and Sambrook, 2012). The gene was amplified by polymerase chain reaction (PCR) with Phusion High-Fidelity DNA Polymerase (Thermo Scientific) following the manufacturer’s recommendations. Genomic DNA from *Pseudomonas aestusnigri* was isolated with the DNeasy, Blood and Tissue Kit (Qiagen GmbH) according to the manufacturer’s protocol and used as template with oligonucleotides PE-H_fw (AGGTCTAGATGGAGGCTACACCTCATG) and PE-H_rv (GTGCTCGAGGTACGGGCAGTTGCCGCGATAATC). The resulting recombinant plasmid pET22b_PE-H_c__6__H_ was used to transform chemical competent *E. coli* DH5α cells (Woodcock et al., 1989) for replication and *E. coli* BL21(DE3) cells (Hanahan, 1983) for T7 DNA polymerase driven expression (Studier and Moffatt, 1986).

#### Recombinant Protein Production

Protein production was carried out in Erlenmeyer flasks filled to 1/10 of the maximal volume with auto induction media (20 g/l tryptone from casein, 5 g/l NaCl, 5 g/l yeast extract, 6 g/l Na_2_HPO_4_, 3 g/l KH_2_PO_4_, 0.6% glycerol, 0.2% lactose, 0.05% glucose) (Studier, 2005) modified as described in^1^ supplemented with 100 μg/ml ampicillin, for 24 h at 30°C with shaking (160 rpm). The culture was inoculated to an optical density of 0.05 (λ = 580 nm) from a culture grown overnight in LB media (Luria/Miller, Carl Roth GmbH & Co. KG) supplemented with 0.5% glucose and 100 μg/ml ampicillin. After the designated production time cells were collected by centrifugation for 30 min at 6,000 × *g*, 4°C, the supernatant was discarded, and cell pellets were stored at −20°C or used subsequently.

#### Protein Purification

Purification of PE-H was performed by immobilized metal ion affinity chromatography (IMAC) and size exclusion chromatography (SEC). Cell pellets were resuspended in lysis buffer (20 mM Na_2_HPO_4_ pH 7.4, 500 mM NaCl, 10 mM imidazole) at 10% (w/v) and disrupted using a high-pressure homogenizer (EmulsiFlex-C5, AVESTIN Europe, GmbH) with three passages at about 8,000 psi. Cell debris and insoluble aggregates were removed by centrifugation (30 min, 4°C, 36,000 × *g*), soluble proteins were mixed with about 5 ml Ni-NTA matrix (Ni-NTA Superflow, Qiagen GmbH) per liter of culture, the matrix was washed and equilibrated with lysis buffer prior to this, and incubated for 30 min at 4°C. The matrix was filled into a gravity flow column and washed with at least 10 column volumes (CV) of washing buffer (20 mM Na_2_HPO_4_ pH 7.4, 500 mM NaCl, 30 mM imidazole) before elution with 3 CV of elution buffer (20 mM Na_2_HPO_4_ pH 7.4, 500 mM NaCl, 500 mM imidazole). Eluted proteins were concentrated by centrifugal ultrafiltration (Vivaspin 20, 10,000 MWCO, Satorius AG) and desalted using 100 mM potassium phosphate buffer pH 7.4 with PD-10 desalting columns (GE Healthcare) according to the manufacturer’s recommendation. Prior to protein crystallization studies, SEC was applied to further improve protein purity. An ÄKTA Explorer system (GE Healthcare) equipped with a HiLoad 16/60 Superdex 200 prep grade column (GE Healthcare) was used and proteins were eluted with 1.5 CV of 10 mM potassium phosphate buffer pH 7.4 as the mobile phase at a flow rate of 1 ml/min. Fractions of 5 ml volume were collected and tested individually for esterase activity with 4-nitrophenyl butyrate as the substrate. Protein containing fractions, as determined by absorption at λ = 280 nm, with esterase activity were pooled, concentrated by centrifugal ultrafiltration, and analyzed or stored at 4°C.

#### Determination of Protein Concentration

Protein concentrations were determined using a micro-volume spectrophotometer (NanoDrop, Thermo Fisher Scientific) using protein specific molecular weight (32,308 Da) and extinction coefficient (48,610 M^–1*^cm^–1^) as calculated using the ProtParam web service (Gasteiger et al., 2005).

#### Sodium Dodecyl Sulfate Polyacrylamide Gel Electrophoresis (SDS-PAGE)

SDS-PAGE analysis was carried out according to Laemmli (1970). Protein containing samples were mixed with sample buffer [50 mM Tris-HCl pH 6.8, 0.03% (w/v) bromphenol blue, 10% (v/v) glycerol, 4% (w/v) SDS, 2% (v/v) 2-mercaptoethanol], boiled for 5–10 min at 98°C, and applied to a 12% polyacrylamide gel using the Mini-PROTEAN system (Biorad GmbH) with Laemmli buffer [25 mM Tris-HCl pH 8.8, 192 mM glycine, 0.1% (w/v) SDS]. After separation for 15 min at 100 V and 40 min at 200 V, staining of the gel with Coomassie solution [10% (w/v) ammonium sulfate; 1.2% (v/v) phosphoric acid (85% aqueous solution), 0.1% Coomassie Brilliant Blue R250, 20% methanol] was applied (Blakesley and Boezi, 1977). Visual documentation was done using an Advanced Imager system (INTAS Science Imaging Instruments GmbH).

### Biochemical Characterization

#### Qualitative Determination of Polyester Hydrolase Activity

Rapid qualitative determination of polyester hydrolase activity was carried out on agar plates containing Impranil DLN-SD (Covestro AG) as the substrate as described earlier (Molitor et al., 2020). 1.5% (w/v) agar-agar (Carl Roth GmbH & Co. KG) was added to either LB media (Luria/Miller, Carl Roth GmbH & Co. KG) for assessment of polyester hydrolase activity of bacteria, or to 100 mM potassium phosphate buffer pH 7.4, for assessment of polyester hydrolase activity of purified enzymes, and sterilized by autoclaving (20 min at 121°C). The molten agar media were allowed to cool down (about 60°C) before addition of heat labile compounds (e.g., antibiotics) and 1% (v/v) Impranil DLN-SD. The media was mixed with a magnetic stirrer, poured into Petri dishes, and dried for 15–30 min under a sterile laminar flow hood (Herasafe KS, Thermo Fisher Scientific). To assess activity, bacterial cells were transferred with a sterile toothpick to the plates and incubated at the respective optimal growth temperature for 1–3 days, or purified enzymes were dissolved in 100 mM potassium phosphate buffer pH 7.4 and directly applied to the plates.

#### Quantitative Determination of Esterase Activity

Esterase activity of purified enzymes was quantified using the substrates 4-nitrophenyl butyrate (*p*NPB) or hexanoate (*p*NPH) as described earlier (Nolasco-Soria et al., 2018). Briefly, 10 μl of enzyme solution was combined with 190 μl substrate solution (1 mM 4-nitrophenyl ester, 5% acetonitrile, 100 mM potassium phosphate buffer pH 7.4) in a flat bottom 96-well microtiter plate and the reaction was followed at 30°C in a microplate reader (SpectraMax i3x, Molecular Devices, LLC) at λ = 410 nm. Initial reaction velocity corrected by a control reaction without enzyme was used to calculate the release of 4-nitrophenol per minute using formula [1].

(1) $$\frac{V\,\left[m\,i\,n^{-1}\right]*v_{m\,t\,p}*F}{d\,\left[c\,m\right]*\varepsilon \,\left[m\,M^{-1}*c\,m^{-1}\right]*v_{e\,n\,z}*c\,\left[m\,g*m\,l^{-1}\right]}=A\,\left[U*m\,g^{-1}\right]$$

V is the initial reaction velocity, v_mtp_ the volume in the well of the microtiter plate, F the dilution factor, d the path length, ε the extinction coefficient of 4-nitrophenol at pH 7.4, v_enz_ the volume of the enzyme sample, c the enzyme concentration and A the specific enzyme activity. One unit (U) was defined as the amount of enzyme needed to release 1 μmol of 4-nitrophenol per minute.

#### Determination of Protein Thermal Melting Point

Protein melting curves were measured by nano differential scanning fluorimetry (nanoDSF) using a Prometheus device (NanoTemper Technologies, Inc.), according to the manufacturer’s recommendation. Briefly, purified enzyme (protein concentration 4–8 mg/ml) in 20 mM Tris pH 8 buffer was loaded into NanoTemper capillary tubes and applied to the Prometheus device for a melting scan at 10% excitation power, from 20 to 95°C at a heating rate of 1°C per minute.

#### Enzymatic Hydrolysis of BHET and PET and Quantification of Reaction Products

For hydrolysis of BHET and PET films, the enzymatic reaction was set up as described earlier (Yoshida et al., 2016) with minor modifications. The reaction mixture in a total volume of 300 μl was composed of 500 nM purified enzyme in 20 mM potassium phosphate buffer pH 7.4 with 20% (v/v) dimethyl sulfoxide (DMSO) and either 0.75 μl 400 mM BHET (95% purity, Sigma Aldrich) dissolved in DMSO or a circular piece of PET film (6 mm diameter). The PET pieces were produced from either amorphous PET film (0.25 mm thickness, Goodfellow Cambridge, Ltd.) or PET film derived from a commercial single use PET water bottle (trademark “Gut und Günstig,” EDEKA) using a puncher, were washed with ethanol p.A., and were dried under a sterile laminar flow hood prior to use. The reaction mixtures were incubated for 24 h for BHET or 48 h for PET film at 30°C. BHET hydrolysis was stopped by removing the enzymes using ultrafiltration with centrifugal filters with a molecular weight cutoff (MWCO) of 10,000 Da (VWR International GmbH). PET film hydrolysis was stopped by heat inactivation of the enzymes for 20 min at 85°C and subsequent filtration with polyamide syringe filters of 0.2 μm pore size. The reaction filtrates were analyzed with an UPLC System (Acquity UPLC, Waters GmbH) equipped with an Acquity UPLC BEH C18 column (1.7 μm particle size) adapted from a published method (Yoshida et al., 2016). The mobile phase consisted of (A) 20 mM Na_2_HPO_4_ pH 2.5 (pH adjusted with H_2_SO_4_) and (B) methanol, the effluent was monitored at λ = 240 nm. The column was kept at constant temperature of 35°C and a flow rate of 0.208 ml/min. The program was 75% (A) and 25% (B) for 1.28 min, followed by a linear gradient to 100% (B) in 2 min, hold 100% (B) for 3 min, linear gradient from 100 to 25% (B) in 1 min and hold 25% (B) until minute 8.28 was reached. For terephthalic acid (TA) and BHET, commercially available standards were used to calculate amounts from calibration curves. For MHET no commercial standard was available. Therefore, a series of enzymatic reactions with BHET as substrate and MHET as major product was performed as described above and the percental distribution of the BHET and the MHET peak areas in combination with the known amount of the substrate BHET was used to calculate a calibration curve for enzymatic hydrolysis of MHET.

### Site Directed Mutagenesis

To introduce single and multiple amino acid substitutions to PE-H, site directed mutagenesis was carried out. Therefore, QuikChange PCR was applied as described earlier (Edelheit et al., 2009) with mutagenic primer pairs (Table 1) and the recombinant plasmid pET22b_PE-H_c__6__H_ as a template. *E. coli* DH5α (Woodcock et al., 1989) cells were transformed with the recombinant plasmids by heat-shock (Hanahan, 1983) for vector replication, plasmid DNA was isolated with innuPREP Plasmid Mini Kit 2.0 (Analytic Jena AG), and mutations were verified by Sanger sequencing (eurofins genomics GmbH and LGC genomics GmbH). Cloning was simulated and sequence analysis was carried out using Clone Manager software (Sci-Ed Software).

**TABLE 1:** Oligonucleotide sequences of primers used for site directed mutagenesis of PE-H.

**Name**	**Sequence (5′->3′)**
S171A	GGCGTCATTGGCTGGGCGATGGGCGGTGGCGGC
	GCCGCCACCGCCCATCGCCCAGCCAATGACGCC
D217A	CTTTGCCTGTGAGTCGGCGGTGATCGCGCCGGTC
	GACCGGCGCGATCACCGCCGACTCACAGGCAAAG
H249A	CAATGGTGGCAGCGCGTACTGCGGTAATGGC
	GCCATTACCGCAGTACGCGCTGCCACCATTG
G254S	CACTACTGCGGTAATAGCGGCAGCATCTACAAC
	GTTGTAGATGCTGCCGCTATTACCGCAGTAGTG
S256N	GCGGTAATGGCGGCAACATCTACAACGATGTG
	CACATCGTTGTAGATGTTGCCGCCATTACCGC
I257S	GGTAATGGCGGCAGCAGCTACAACGATGTGCTG
	CAGCACATCGTTGTAGCTGCTGCCGCCATTACC
Y258N	GTAATGGCGGCAGCATCAACAACGATGTGCTGAGC
	GCTCAGCACATCGTTGTTGATGCTGCCGCCATTAC
N259Q	GGCGGCAGCATCTACCAGGATGTGCTGAGCCGG
	CCGGCTCAGCACATCCTGGTAGATGCTGCCGCC
ext.loop	GCAGCCACTACTGCGGTAATTCGGGCAACTCGAATCAGGATG
	CCGAACCGGCTCAGCACATCCTGATTCGAGTTGCCCGAATTAC
Y250S	AATGGTGGCAGCCACTCCTGCGGTAATGGCGGC
	GCCGCCATTACCGCAGGAGTGGCTGCCACCATT
Q294A	CACACTTCCGACTCTGCCATCTCCGATTATCGC
	GCGATAATCGGAGATGGCAGAGTCGGAAGTGTG
I219Y	GTGAGTCGGATGTGTACGCGCCGGTCCTCCAG
	CTGGAGGACCGGCGCGTACACATCCGACTCACA

### Crystallization

#### Wild Type Enzyme PE-H

Several crystals were observed by using commercial kits from NeXtal (Qiagen, Hilden, Germany) and Molecular Dimensions (Suffolk, England) for initial screening. 0.1 μL homogenous protein PE-H (11 mg/ml, in 10 mM potassium phosphate buffer pH 7.4) was mixed with 0.1 μL reservoir solution and equilibrated against 40 μL reservoir solution in sitting drop MRC3 plates (Swissci) at 12°C. Crystals or needles appeared with this vapor diffusion method after a few days.

Variation of one of these conditions [0.1 M sodium acetate pH 4.5, 16% (w/v) PEG 3000] via grid screen (sitting drop, 1 μl + 1 μl over 300 μl reservoir at 12°C) resulted in well diffracting crystals with a maximum size of 120 × 30 × 20 μm after 1 week in an optimized condition composed of 0.1 M sodium acetate pH 4.5, 16% (w/v) PEG 3000, 0.036 mM LysoFos Choline14.

#### Enzyme Variant PE-H Y250S

Initial screening was performed as for the wild type (WT) PE-H (14 mg/ml of PE-H Y_250_S). Within 3 weeks, rod shaped crystals appeared and reached their maximum size of 75 × 35 × 20 μm in 0.2 M lithium sulfate, 0.1 M sodium citrate pH 3.5 and 28% (v/v) PEG 400.

To cryoprotect the crystals, all drops were overlaid with 2 μl mineral oil before the crystals were harvested and flash frozen in liquid nitrogen.

### Data Collection and Structure Determination

Data sets of a single crystals of the wild type enzyme PE-H were collected at the ID29 at ESRF (Grenoble, France) at 100K equipped with a Dectris Pilatus 6M detector. Data sets for enzyme variant PE-H Y250S were collected at P13 at DESY (Hamburg, Germany) at 100K equipped with a Dectris Pilatus 6M detector.

Data sets were processed with XDS (Kabsch, 2010). For PE-H, the XDS_ASCII.HKL-file together with the protein sequence was used as input in autorickshaw webservice^2^ to obtain initial phases via molecular replacement. These output files were directly used in ARP/wARP webservice^3^ for further model building and phase improvement. Subsequently, the model was further built and refined manually using COOT (Emsley et al., 2010) software followed by REFMAC5 from the ccp4 suite (Collaborative Computational Project, 1994). For PE-H Y250S, the refined wild type structure served as search model in molecular replacement. Further model building and refinement was performed as already described. Structures were deposited in the protein data bank^4^ under the accession codes 6SBN (WT PE-H) and 6SCD (PE-H Y250S). All structure related figures were prepared with PyMOL (Schrödinger, LLC, United States)^5^, for the structure based alignments we used the PDBeFold webserver^6^.

### Molecular Docking Computations

For the molecular docking, ligands BHET, MHET, and 2-HE(MHET)_4_ were drawn and converted into a 3D structure with Maestro (Schrödinger, LLC, New York). The ligands and proteins were protonated according to pH 7.4 using the Epik routine in Maestro. The ligands were subsequently docked into the binding pocket of the respective enzymes using a combination of AutoDock as a docking engine and the DrugScore^2018^ distance-dependent pair-potentials as an objective function (Goodsell et al., 1996; Sotriffer et al., 2002; Dittrich et al., 2019). In the docking, default parameters were used, with the exception of the clustering RMSD cutoff, which was set to 2.0 Å. Binding modes were considered valid, if they were part of a cluster that comprised at least 20% of all docking poses.

### Bioinformatic Tools and Software

Multiple sequence alignment was carried out with Clustal Omega using default settings (Sievers et al., 2011), basic local alignment searches (BLAST) were done using the web service of the National Center for Biotechnology Information (NCBI) (Altschul et al., 1990; Wheeler et al., 2003), for data visualization, GraphPad Prism (GraphPad Software, Inc., United States) and OriginLab (OriginLab Corporation, United States) were used. For the identification and description of molecular cavities, the MOLE 2.5 software (Sehnal et al., 2013) was used employing default options with an 8.0 probe radius.

## Results

### PE-H From *Pseudomonas aestusnigri* Is a Polyester Hydrolase

**FIGURE 1:**
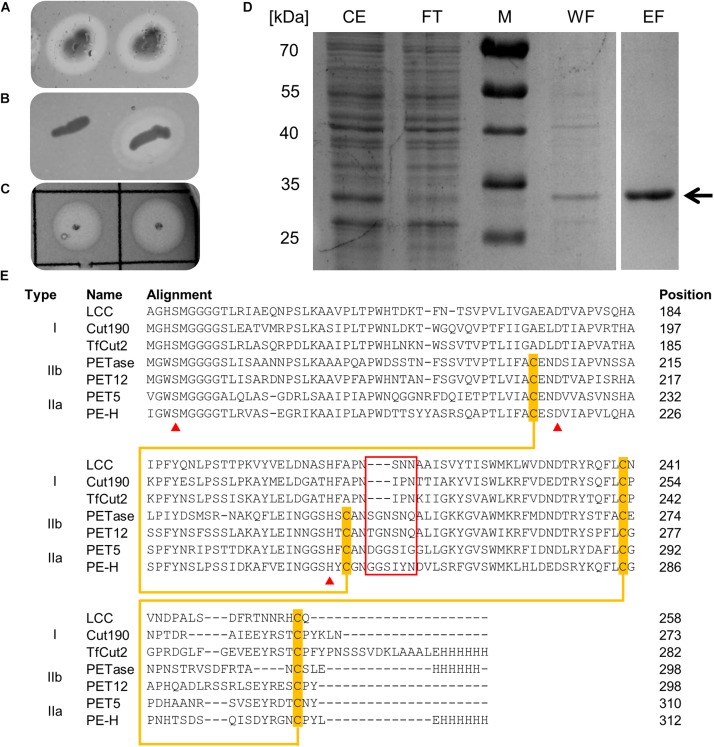
**(A)** Colonies of *P. aestusnigri* grown on an Impranil DLN containing agar plate show clearing halos, indicating polyester hydrolase activity. **(B)** Hydrolytic activity of *E. coli* BL21(DE3) empty vector control (left) and PE-H production strain (right) on Impranil DLN containing agar plate. **(C)** Hydrolytic activity of purified PE-H on Impranil DLN containing agar plate. **(D)** Coomassie brilliant blue stained gel after SDS-PAGE of cell extracts of *E. coli* BL21(DE3) containing plasmid pET22b_PE-H_c__6__H_ before and after purification by IMAC; the position of PE-H is indicated by an arrow. Lanes contained cell extract (CE), flow through (FT), washing step (WF), and eluted protein (EF); the size of the molecular weight standards (M) are indicated on the left. **(E)** Part of a multiple sequence alignment of PE-H with amino acid sequences of different PET hydrolytic enzymes using the program Clustal Omega. The full length alignment can be found in the Supplementary Figure S1. The enzymes were assigned to different types of polyester hydrolases (Joo et al., 2018). Amino acid residues of the catalytic triad are marked by a red triangle, disulfide forming cysteine residues are highlighted in orange and connected by an orange line. Amino acids forming the extended loop region which is specific for type II PET hydrolytic enzymes are framed in red. Abbreviations are: leaf-branch compost metagenome cutinase (LCC); *Saccharomonospora viridis* cutinase (Cut190); *Thermobifida fusca* cutinase (TfCut2); *Ideonella sakaiensis* PET hydrolase (PETase); *Polyangium brachysporum* PET hydrolase (PET12); *Oleispira antarctica* PET hydrolase (PET5).

*Pseudomonas aestusnigri*, a bacterium belonging to the *P. pertucinogena* phylogenetic lineage, shows hydrolytic activity on different polyester substrates (Molitor et al., 2020). Additionally, we observed hydrolytic activity indicated by formation of clear halos upon growth of *P. aestusnigri* on agar plates containing Impranil DLN, an anionic aliphatic polyester-polyurethane used for surface coating of textiles (Figure 1A). Bioinformatic analysis of the *P. aestusnigri* genome sequence (Gomila et al., 2017) led us to predict the existence of a polyester hydrolase coding gene (Bollinger et al., 2020). The respective gene was cloned into a pET-22b(+) expression vector and produced by expression in *E. coli* BL21(DE3). The recombinant bacteria showed significant hydrolytic activity when grown on Impranil DLN containing solid media (Figure 1B). Hence, the predicted polyester hydrolase gene indeed codes for a functional polyester hydrolase which we named PE-H. The respective gene (locus tag: B7O88_RS11490) codes for a protein of 304 amino acids (protein id: WP_088276085.1) comprising a signal peptide of 25 amino acids for Sec-dependent translocation as predicted by SignalP (Almagro Armenteros et al., 2019). For further characterization, the protein was produced in soluble form along with its native signal peptide and fused to the vector-encoded hexa-histidine tag, which allowed a one-step purification by immobilized metal ion chromatography (IMAC) (Figure 1D). The purified protein has a molecular weight of about 32 kDa and prominent activity toward the polymer substrate Impranil DLN (Figure 1C). Furthermore, a basic local alignment search using BLAST (Altschul et al., 1990; Wheeler et al., 2003) with the protein sequences in the protein database (PDB) as search set revealed similar sequences of cutinases and PET hydrolytic enzymes originating from *S. viridis*, *Thermobifida* sp. and *I. sakaiensis* with 48–51% identity at more than 80% query coverage.

The relation of PE-H to cutinases and other PET hydrolytic enzymes was analyzed by multiple sequence alignments of the amino acid sequence of PE-H with representative examples of each type of proven PET hydrolytic enzymes as proposed by Joo et al. (2018) was done using Clustal Omega. For type I, sequences, the leaf-branch compost metagenome cutinase LCC (Sulaiman et al., 2012), the cutinase Cut190 from *S. viridis* (Kawai et al., 2014), and the cutinase TfCut2 from *T. fusca* (Roth et al., 2014) were used. PETase from *I. sakaiensis* (Yoshida et al., 2016) and PET12 from *Polyangium brachysporum* (Danso et al., 2018) served as examples for type IIb and PET5 from *Oleispira antarctica* (Danso et al., 2018) for type IIa, as demonstrated before (Taniguchi et al., 2019). The alignment revealed a clear discrimination of PE-H from cutinases of type I, owing to an additional disulfide bond and additional amino acids close to the catalytically active histidine (Figure 1E and Supplementary Figure S1). Both characteristics are typical for PET hydrolytic enzymes of type II (Joo et al., 2018). Furthermore, due to the amino acid composition of the region constituting additional amino acids, known as extended loop in case of PETase (Joo et al., 2018), PE-H can be classified as a type IIa PET hydrolytic enzyme together with PET5 from *Oleispira antarctica* (Danso et al., 2018). In fact, PE-H is a close homolog of the enzymes encoded by *Pseudomonas sabulinigri*, *P. pachastrellae*, and *P. litoralis* [all belong to the phylogenetic lineage of *P. pertucinogena* (Peix et al., 2018)], which were proposed as PET-degrading enzymes of type IIa by *in silico* sequence comparison (Joo et al., 2018).

### PE-H Degrades PET

**FIGURE 2:**
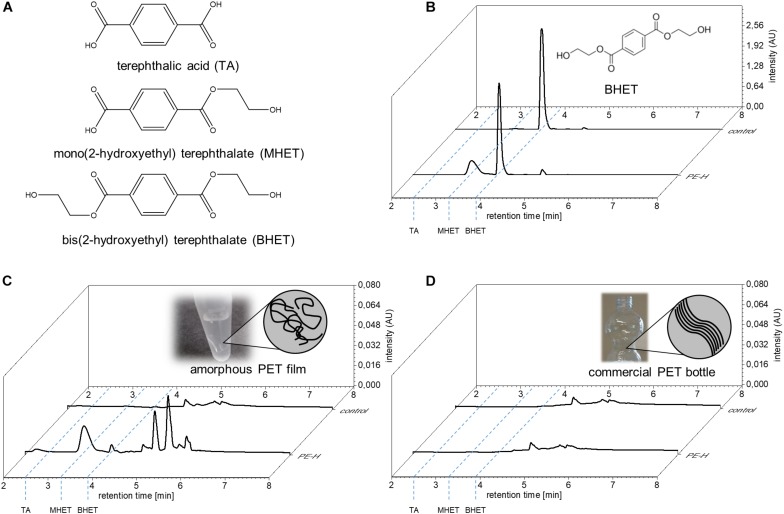
Hydrolysis of PET by PE-H. **(A)** Structural formula of aromatic reaction products obtained by hydrolysis of PET or BHET. **(B)** UPLC analysis of reaction products obtained with PE-H and the substrates bis(2-hydroxyethyl) terephthalate (BHET), **(C)** amorphous PET film (PETa) and **(D)** PET film derived from a commercial single use PET bottle (PETb). The UPLC trace at 240 nm wavelength is shown for the control reaction without addition of the enzyme (trace in the back, labeled as control), and the enzyme reaction (trace in front, labeled PE-H). The retention times of the standard compounds terephthalic acid (TA), and bis(2-hydroxyethyl) terephthalate (BHET) as well as of the reaction product mono(2-hydroxyethyl) terephthalate (MHET) are indicated by blue dashed lines. The respective substrate of the reaction is depicted in the back of each diagram. For BHET, the structural formula is given; for PET films, a cartoon representation is shown to illustrate the different arrangement of PET fibers in amorphous and crystalline films.

The classification of PE-H as a type IIa PET degrading enzyme suggested PET degrading activity. Therefore, we tested the enzymatic activity of PE-H with the substrates monomeric bis(2-hydroxyethyl) terephthalate (BHET), amorphous PET film (PETa) and PET film derived from a commercial single use PET bottle (PETb). The PET polymer consists of esterified terephthalic acid (TA) and ethylene glycol (EG), allowing for TA, EB, and esters of both compounds with different degree of polymerization as degradation products, for example BHET or mono(2-hydroxyethyl) terephthalate (MHET) (Figure 2A). The enzyme hydrolyzed both BHET (Figure 2B) and PETa (Figure 2C), releasing mono(2-hydroxyethyl) terephthalate (MHET), but no terephthalic acid (TA). When PETb was used as a substrate, no hydrolysis product was detected (Figure 2D).

The determination of the crystal structure of PETase from *I. sakaiensis* (Yoshida et al., 2016) by different groups in 2017/2018 (Han et al., 2017; Austin et al., 2018; Chen et al., 2018; Fecker et al., 2018; Joo et al., 2018; Liu B. et al., 2018; Liu C. et al., 2018; Palm et al., 2019) allowed for detailed insights into the structure-function relationship of PETase with regard to PET degradation. By structural comparison to cutinases, the active site cleft of PETase was shown to be wider and shallower which seemed to be important for the PET hydrolytic activity of PETase (Austin et al., 2018; Liu B. et al., 2018). Moreover, a number of regions on the protein surface were proposed to be important for the efficient PET hydrolysis, among them (i) serine residue S238 which showed an important contribution to the enzyme activity (Joo et al., 2018), (ii) tryptophan residue W159 with proposed contribution in π-stacking interaction with terephthalic acid moiety of PET (Han et al., 2017; Chen et al., 2018), and (iii) a conserved extended loop region consisting of six amino acids connecting β8-α6 (Joo et al., 2018), which was proposed to mediate substrate binding. The amino acid composition of PE-H differs at the corresponding positions (Supplementary Table S1) prompting us to introduce a series of single amino acid substitutions into PE-H by site directed mutagenesis and subsequently evaluate the activity of the respective enzyme variants with different substrates.

**FIGURE 3:**
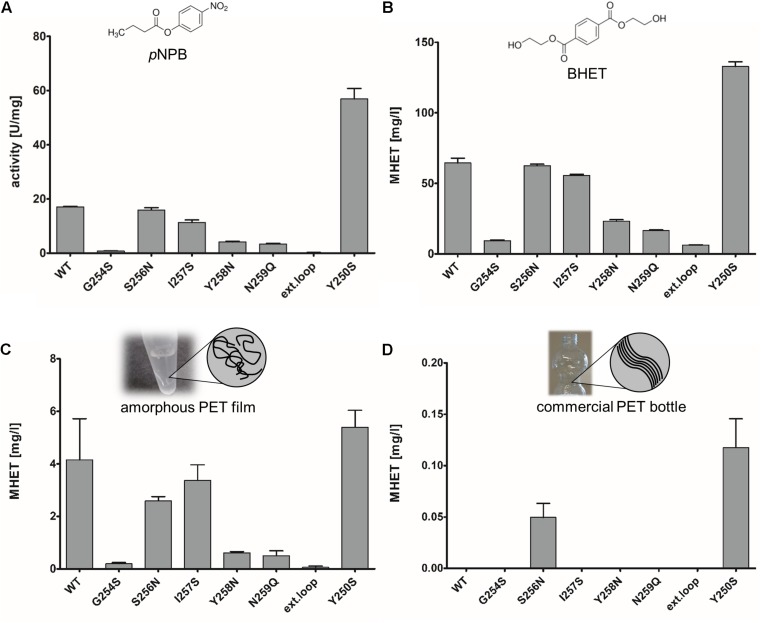
Enzymatic activity of PE-H and different variants constructed by site directed mutagenesis. Substrates were **(A)** 4-nitrophenyl butyrate (*p*NPB), **(B)** bis(2-hydroxyethyl) terephthalate (BHET), **(C)** amorphous PET film, and **(D)** PET from a commercial single use bottle. Standard deviations of three individual reactions are shown as error bars. The respective substrate is depicted above each diagram. For *p*NPB and BHET, structural formulas are shown; for amorphous and commercial PET a cartoon representation is shown to illustrate the different arrangement of PET fibers.

The substitution of active site amino acids by alanine led to inactive variants as expected (data not shown). The extended loop region can be found for PE-H as well, with an amino acid composition representative for polyester hydrolases of type IIa (Joo et al., 2018). Both, single substitutions at each position of the extended loop to those present in PETase, and the exchange of the entire extended loop region to that of PETase were constructed and the resulting variants were produced, purified, and tested for activity against the substrates 4-nitrophenyl butyrate (*p*NPB), BHET, and PET film. In comparison to the wild type enzyme PE-H, neither the single substitution variants nor the loop exchange variant, designed to match the amino acid residues at the corresponding positions of PETase (Supplementary Table S1), showed a higher specific activity toward BHET or PET film. Of these variants, G254S, Y258N, N259Q, and the variant with the combined mutations also showed a significantly decreased esterase activity determined with *p*NPB as the substrate (Figure 3A). The reduced activity of the variants was accompanied by a decrease in melting temperature of about 5–10°C (Supplementary Table S2) in comparison to the wild type enzyme (T_m_ at about 51°C), indicating a destabilizing effect of the mutations. Variant PE-H S256N, I257S, and Y250S showed a less drastic decrease in melting temperature of about 1–3°C.

Interestingly, the variant Y250S of PE-H showed a more than threefold increase in specific activity for *p*NPB, assuming a general importance of this amino acid position for enzymatic activity. The same trend was observed with BHET as the substrate (Figure 3B), showing decreased activity of all variants compared to the wild type except for variant Y250S. Similar results were obtained with PETa as the substrate with variant Y250S producing more MHET than the wild type enzyme or any other variant tested (Figure 3C). Nevertheless, the effect was less clear compared to the soluble substrates BHET and *p*NPB. The activity of the enzyme on PET derived from a commercial single use bottle as determined by MHET release appeared to be in general low. Only two variants, Y250S and S256N, led to detectable formation of MHET from PETb, but only at low amounts (Figure 3D).

### PE-H Crystal Structure Confirms Similarity to PET Degrading Enzymes

**FIGURE 4:**
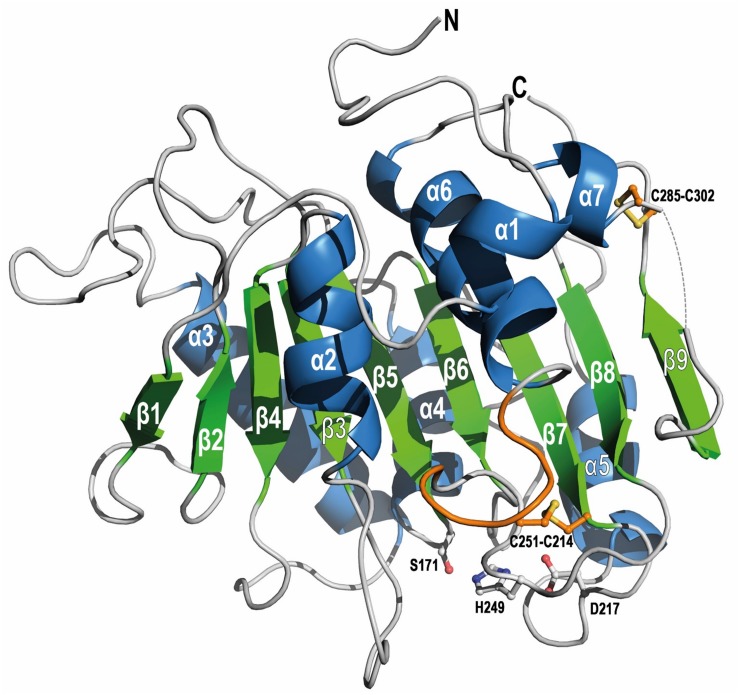
Overall fold of WT PE-H (PDB code 6SBN) in cartoon representation. The short stretch (aa 286–291) is not visible (gray dashed line). The α/β-fold consists of a central twisted β-sheet composed of 9 β-strands flanked by 7 α-helices on both sides. The extended loop region and the Cys residues (ball-and-sticks) forming the disulfide bonds are highlighted in orange. Residues building the catalytic triad are shown as gray ball-and-sticks with labels. Rotations between the structure depictions are indicated in Supplementary Figure S4.

In order to gain insight into the molecular basis of the polyester hydrolytic activity of PE-H and its variant Y250S, we solved the crystal structures of both enzymes. The PE-H structure was solved at 1.09 Å resolution, containing one monomer in the asymmetric unit, with 10.7% for R_work_ and 13.7% for R_free_. Although the electron density was of extremely good quality, the N-terminal part (aa 1–37) and one short stretch (aa 286–291) were not visible. The structure of variant PE-H Y250S was solved at 1.35 Å resolution; here, only the N-terminus (aa 1–39) is missing. Electron densities around the active site of PE-H and variant Y250S are shown in Supplementary Figure S2. The crystal structure of PE-H Y250S contained a PEG molecule bound to the protein surface (Supplementary Figure S3). Data collection and structure refinement statistics are given in the Supplementary Material (Supplementary Table S3). The PE-H protein shows a canonical α/β-fold consisting of a central twisted β-sheet composed of 9 β-strands flanked by 7 α-helices on both sides (Figure 4), as already reported for homologous structures, i.e., cutinases and PETases (Han et al., 2017; Numoto et al., 2018). Two disulfide bonds are present in PE-H connecting C214-C251 and C285-C302 as is common for type II PET degrading enzymes.

**FIGURE 5:**
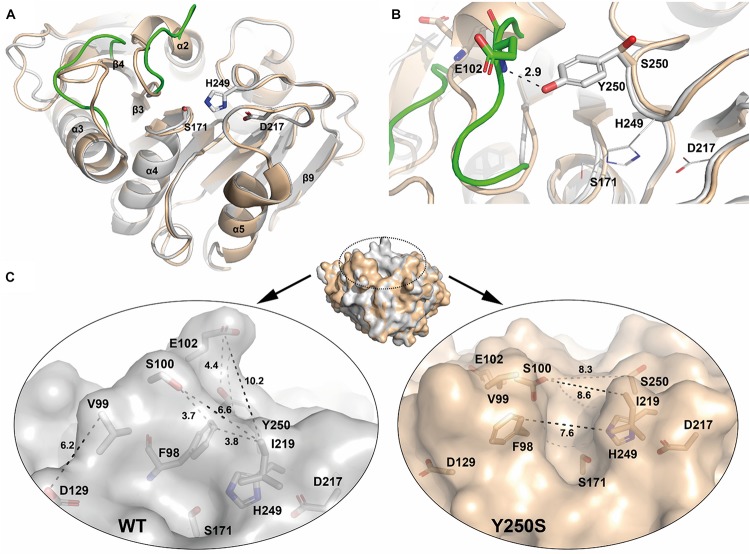
Superposition of PE-H (PDB code 6SBN, silver) and its variant Y250S (PDB code 6SCD, wheat). **(A)** The top view on the active site and the different orientation of the two loops is shown. Residues of the catalytic triad are depicted as sticks with labels, loops of PE-H are colored in green to highlight the structural differences. **(B)** A zoom in the active site cleft with the catalytic residues depicted as lines. Remarkably, Y250 (gray sticks) makes a polar contact with E102 (green sticks) in the wild type protein, the distance is shown as a dashed line and given in Å, whereas in variant Y250S, residue E102 (wheat sticks) is far apart [color code as in **(A)**]. **(C)** Surface representation of WT PE-H (silver) and variant Y250S (wheat), on top, both structures are superposed with the catalytic cleft indicated by a dashed circle. In the panels, the differences in both structures are displayed in detail; with WT PE-H in the left panel and variant Y250S in the right panel. Residues confining the active site cleft are depicted as sticks, labeled, and connected by dotted lines, with the corresponding distances given in Å. Rotations between the different structure depictions are indicated in Supplementary Figure S4.

Both structures reported here display a nearly identical overall fold (WT PE-H to PE-H Y250S: rmsd 0.188 Å over 217 Cα atoms) with main differences in two loop regions: the loop connecting β3-α2 (aa 98–104) adopts a “close” conformation in WT PE-H with regard to the active site cleft, with a loop connecting β4-α3 (aa 123–128) positioned parallel to it, while both are shifted against each other in the Y250S mutant (Figure 5A), thereby creating more space in the catalytic site. The highly conserved residues S171, D217 and H249 build the catalytic triad which is located closely below the surface with the S171 position known as the “nucleophilic elbow”. The oxyanion hole is constituted by the backbone NH groups of M172 and F98. The loop arrangement narrowing the active site cleft in the wild type enzyme is stabilized by a polar contact between the hydroxyl group of Y250 and the backbone amine of E102 (Figure 5B). This structural rearrangement further leads to an increased active site cavity volume from 153 Å^3^ for WT PE-H to 362 Å^3^ for variant Y250S as determined with the program MOLE2.5. A comparison of the active site cleft molecular surface of WT PE-H and variant Y250S shows the altered topology affecting the observed increase in active site cavity volume. In WT PE-H, residues of the loop connecting β3-α2 (F98, V99, S100) together with D129 and I219 are arranged in a way that access to the active site is limited, whereas PE-H variant Y250S shows a much deeper cleft (Figure 5C).

To identify structural homologs of PE-H, we performed structural alignments of both PE-H structures independently against the PDB. At maximum, we found 60 similar protein chains with rmsd values in the range of 1.1 to 2.7 Å for Cα atoms and 53% sequence identity at maximum (data not shown). For PE-H, the Cut190 triple mutant (TM) S176A/S226P/R228S (PDB 5ZRR) from *S. viridis* is the most similar (rmsd 1.18 Å); for variant Y250S, it is the PETase double mutant (DM) R103G/S131A (PDB 5XH3) from *I. sakaiensis* (rmsd 1.17 Å). The 10 protein chains most similar to both PE-H structures described here are listed in Supplementary Table S4. To further analyze the different architecture of both PE-H variants reported here, we compared their surfaces with those of their structurally most similar homolog (Supplementary Figure S5). All four molecules share a similar pattern of surface charge which is dominated by larger patches of either positive or negative charge or hydrophobic areas, respectively. With regard to the active site cleft, Cut190 TM shows a similar narrowed active site as does WT PE-H, whereas PETase DM has a larger and deeper cleft as is the case for variant Y250S.

**FIGURE 6:**
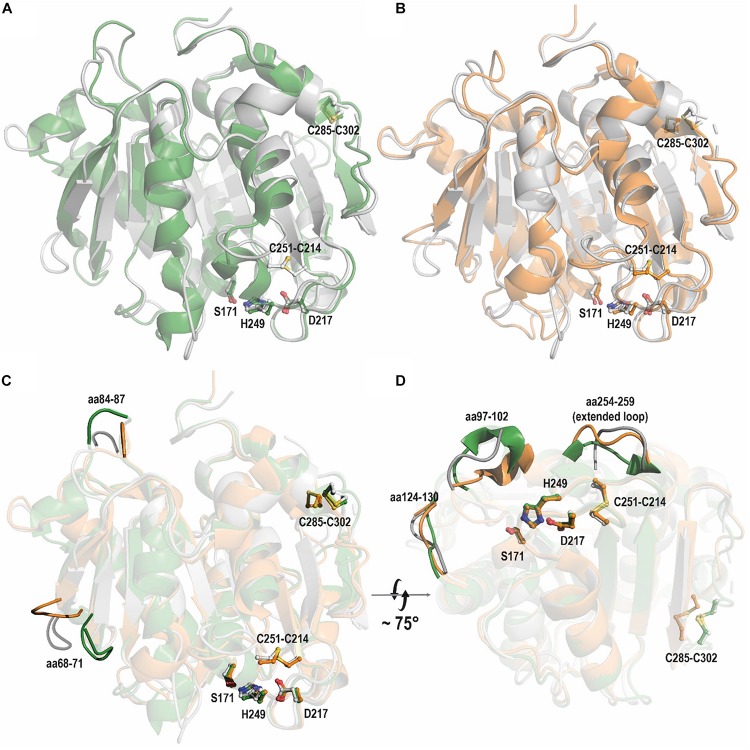
Overlay of PE-H WT (PDB code 6SBN, gray) with **(A)** Cut190 (PDB code 4WFI, green) and **(B)** PETase (PDB code 6ANE, orange). Residues of the active site and disulfide bonds are represented as sticks with labels (according to PE-H numbering). **(C)** Superimposition of PE-H (PDB code 6SBN, gray), PETase (PDB code 6ANE, orange), and Cut190 (PDB code 4WFI, green). Five regions of the superimposition are represented in full tone on fade-out color to highlight the differences of the structures. In **(D)** the structures are turned from bottom to top.

Superimposition of the PE-H structure and the structures of Cut190 (Figure 6A) and PETase (Figure 6B) revealed a high structural identity. PE-H possesses a disulfide bond linking residues C285 and C302 which is also present in Cut190, and a second disulfide bond located close to the active site (Figure 6A, C251-C214); both these disulfide bonds are also present in PETase (Figure 6B). These three structures which represent PET hydrolytic enzymes of types I, IIa, and IIb differ mainly within five loop regions (Figures 6C,D) comprising PE-H amino acid positions 68–71, 84–87, 97–102, 124–130, and 254–259. Three of these regions at positions 97–102, 124–130, and 254–259 are located in the vicinity of the active site indicating a putative contribution to substrate binding.

### Molecular Docking Computations Suggest PE-H Substrate Binding Mode

Substrate binding was analyzed by docking the PET tetramer 2-HE(MHET)_4_, BHET, and MHET to wild type PE-H and variant Y205S using predicted protonation states at pH 7.4 for both the ligands and proteins. Lowest-energy configurations of the ligands in the proteins from the largest cluster were taken as binding poses as done previously (Diedrich et al., 2016; Krieger et al., 2017). Binding modes were considered valid, if at least 20% of all poses are contained in this cluster. During the docking, ligands were allowed to explore the whole protein to ensure an unbiased sampling of potential binding poses.

**FIGURE 7:**
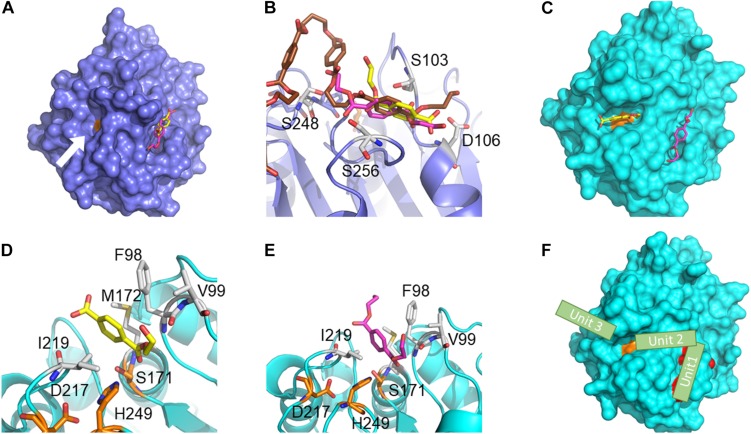
Predicted ligand binding modes in wild type PE-H and variant Y250S. The predicted binding poses of BHET (magenta), MHET (yellow), and 2-HE(MHET)_4_ (brown) in WT PE-H (navy) and the variant Y250S (cyan). In **(A,C,F)** S171 is shown in orange, and in **(B,D,E)** the catalytic triad (S171, D217, and H249) is shown in orange and interacting residues are shown in white. **(A)** In wild type PE-H, BHET and MHET bind to a groove adjacent to the catalytic site (white arrow). **(B)** BHET and MHET bind to the hydrophobic groove and are stabilized by hydrogen bonding interactions with S103, D106, S248, and S256. 2-HE(MHET)_4_ binds similarly to BHET and MHET in the groove adjacent to the catalytic site. **(C)** In the variant Y250S, MHET binds to the catalytic site, while BHET occupies the hydrophobic groove. **(D)** MHET binds to the catalytic site and is stabilized by hydrophobic interactions to F98, V99, M172, and I219 such that S171 can attack the carbonyl carbon for ester hydrolysis. **(E)** A second binding pose of BHET binds similar to MHET. **(F)** Proposed mechanism of PET polymer interaction. Residues G254, Y258, and N259, which when substituted decrease esterase activity, are shown in red. One polymer unit (stylized green rectangle) binds to the groove adjacent to the catalytic site, a second unit bridges the distance to the catalytic site, and a third unit is cleaved from the polymer chain at the catalytic unit.

The predicted binding poses provide mechanistic insights into the function of PE-H and highlight the differences between wild type PE-H and variant Y250S. In PE-H, MHET and BHET are predicted to bind adjacent to the catalytic site (Figure 7A). In the *apo* crystal structure, the catalytic site of PE-H is apparently too narrow to allow favorable substrate binding (Figure 5C). This suggests that in PE-H a conformational change is necessary to accommodate a substrate. BHET and MHET bind with the phenyl rings to a hydrophobic groove and are additionally stabilized via hydrogen-bonding interactions to S103, D106, S248, and S256 (Figure 7B). Although no valid binding mode was identified for 2-HE(MHET)_4_, as no cluster contained more than 4% of all binding poses, the lowest-energy pose found in this docking is located in the adjacent groove like BHET and MHET (Figure 7B), which hints at the groove’s importance for the function of PE-H.

In the variant Y250S, the catalytic site has twice the volume than in wild type PE-H. As a result, the predicted binding pose of MHET is located in the catalytic site, whereas BHET still binds to the same groove as in PE-H (Figure 7C). MHET is stabilized by hydrophobic interactions to F98, V99, M172, and I219, and the ester carbonyl carbon is placed at an optimal distance to be attacked by S171 (Figure 7D). A second binding pose of BHET, which is 0.43 kcal⋅mol^–1^ less favorable than the lowest-energy one, is found in the catalytic site similar to MHET (Figure 7E) and is thereby in an optimal position to be hydrolyzed by S171. Again, no valid binding modes were identified for 2-HE(MHET)_4_, likely owing to the size of the ligand. The presence of binding poses for BHET adjacent to the active site suggests a possible polymer binding mode, where one polymer unit binds to the groove adjacent to the catalytic site, a second unit bridges the distance to the catalytic site, and a third unit is cleaved from the polymer chain (Figure 7F).

## Discussion

Enzymes acting on PET are almost exclusively homologs of cutinases with many of them originating from thermophilic actinobacteria (Nikolaivits et al., 2018). PETase from *I. sakaiensis* was the first enzyme isolated from a mesophilic host and catalyzing the biodegradation of PET at mild temperature of 30°C (Yoshida et al., 2016). Structural studies showed characteristic features discriminating PETase from cutinases with PET hydrolytic activity (Joo et al., 2018) and lead to the proposal of three subclasses of PET hydrolyzing enzymes. With this information, novel PET hydrolytic enzymes were identified, among them enzymes from marine origin (Danso et al., 2018). This finding is of considerable interest because the oceans are known to be a sink for the worldwide plastic waste (Lebreton et al., 2018).

### A Novel PET Degrading Enzyme From the Marine Bacterium *P. aestusnigri*

In this study, we have identified the protein PE-H, a novel polyester hydrolase from the marine bacterium *P. aestusnigri* which hydrolyses different polyethylene terephthalate substrates with mono(2-hydroxyethyl) terephthalate (MHET) as the major hydrolysis product. The formation of MHET, but not TA as hydrolysis product was previously described for other PET degrading enzymes, for example TfCut2 from *T. fusca* (Barth et al., 2015; Wei et al., 2016), Thc_Cut2 from *T. cellulosilytica* (Herrero Acero et al., 2011), and PETase from *I. sakaiensis* (Yoshida et al., 2016). However, in case of TfCut2, MHET was almost completely hydrolyzed to TA after 24 h of reaction (Barth et al., 2015), and the cutinases Thc_Cut1 from *T. cellulosilytica* and Thf42_Cut1 from *T. fusca* were reported to release more TA than MHET from PET film after a reaction time of 120 h (Herrero Acero et al., 2011). Hence, PE-H accumulating MHET from PET film appears to be common feature among PET hydrolyzing enzymes, but the almost complete absence of TA after 48 h of reaction was not observed before.

Hydrolysis of PET by PE-H yielded 4.2 (±1.6) mg/L MHET after a reaction time of 48 h at 30°C, variant PE-H Y250S produced 5.4 (±0.6) mg/L MHET under the same conditions. This amount is considerably lower than reported for the *I. sakaiensis* PETase with more than 150 mg/L after 96 h reaction time at 30°C (Austin et al., 2018). However, it should be mentioned that activities are difficult to compare because different studies use PET substrates which differ among each other, e.g., by the degree of crystallinity. Obviously, a comprehensive comparative study of different PET degrading enzymes would be required. In case of PE-H, the marine origin of the producer *P. aestusnigri* suggests that natural substrates may be aliphatic polyesters produced by algae and plants, because marine bacteria of the *P. pertucinogena* lineage have been found associated with these organisms (Bollinger et al., 2020).

### Rational Mutagenesis Resulted in PE-H Variant Y250S With Improved Activity

Wild type PE-H was unable to hydrolyze a PET film substrate obtained from a commercial PET bottle (PETb). This observation prompted us to identify differences in the amino acid sequences between PE-H and the *I. sakaiensis* PETase and analyze them by a site directed mutagenesis study. As the result, a single amino acid substitution (Y250S) was identified which led to a significant increase in enzymatic activity of PE-H toward different substrates, including PETb. In comparison to the results obtained with PETa, the best performing variant PE-H Y250S showed a 45-fold reduced amount of MHET produced with PETb as the substrate. This observation can be explained by the molecular arrangement of the PET fibers in PETb which is known to comprise more crystalline regions and thus, is more recalcitrant to enzymatic degradation (Wei and Zimmermann, 2017). The highly ordered structure of crystalline PET hampers the enzymes access to single PET fibers, particularly at temperatures below the glass transition temperature of the polymer. Nevertheless, two single amino acid substitution variants, S256N and Y250S, produced detectable amounts of MHET from PETb while the wild type enzyme did not.

### The Crystal Structure of PE-H Is the First Structure of Type IIa PET Hydrolytic Enzyme

In an attempt to rationalize the PE-H activity toward PET as a substrate and to explain the effect of the Y250S substitution, we report here the first crystal structure of a type IIa PET hydrolytic enzyme and its comparison with the crystal structure of the improved variant PE-H Y250S. Interestingly the comparison of both structures showed that the substitution Y250S replacing the aromatic residue tyrosine located next to the active site histidine by the small residue serine significantly improved the enzymatic activity toward different substrates. The relevance of this serine residue has also been observed with PETase variant S238F which showed an about 40% reduced enzymatic activity (Joo et al., 2018). Thus, a small, polar, and uncharged amino acid at the position next to the active histidine seems to be an important characteristic of PET hydrolytic enzymes of type IIb, whereas enzymes of type I and type IIa possess large aromatic residues at the corresponding position (see Figure 1E and Supplementary Figure S1). This finding is also supported by a very recent study which reported a significant increase in activity of the cutinase TfCut2 from *Thermobifida fusca* obtained by substitution of F209 (the corresponding position in PE-H is Y250) with serine and alanine (Furukawa et al., 2019). The authors tested additional substitutions at this position introducing amino acids with chemically different side-chains and concluded that the increase in activity correlated with the size of the respective side-chain. The structural analysis of PE-H and the variant Y250S showed a rearrangement of the loop connecting α2-β3, creating space which results in a better accessible active site. The role of the active site cleft size was also found to be important for PETase, which was reported to be three times wider at its widest point compared to *T. fusca* cutinase TfCut2 (Austin et al., 2018) and also wider compared to *Fusarium solani pisi* cutinase (Liu B. et al., 2018). Hence, the authors proposed that the wider active side cleft enables PETase to accommodate larger substrates like PET. The enhanced activity of PETase at lower temperatures compared to cutinases was attributed to enhanced flexibility within the active center whereby the active site proximal disulfide bridge diminishes loss of stability that is to be expected as tradeoff for enhanced flexibility (Fecker et al., 2018). This disulfide bridge is a common feature of all type II PET hydrolytic enzymes and is in consequence also found in PE-H. The reshaping of the active site cleft of PE-H may therefore be the main reason for the enhanced catalytic activity observed in this study, enabling the enzyme to accommodate larger substrates as seen with PET from a commercial single use PET bottle. In PE-H, besides the steric effects of a larger amino acid, a polar contact between Y250 and E102 was observed which may contribute to narrowing the active site. However, most cutinases carry a phenylalanine at the corresponding position (Joo et al., 2018) which does not allow for a polar contact. This observation therefore does not explain the differences between PET hydrolytic enzymes of type I and II regarding PET hydrolysis. To reveal further differences between PET hydrolytic enzymes of types I, IIa, and IIb, the structures of one enzyme of each type (namely PE-H, Cut190, and PETase) were superimposed and three loop regions located close to the active site were identified as differing. Some amino acids of the corresponding regions in PETase were already reported to be involved in substrate interaction, for example Y87 (F98 in PE-H) and N246 (Y258 in PE-H) which were assigned to substrate binding subsites I and IIc (Joo et al., 2018), respectively.

### The PET Binding Mode of PE-H

To gain insight into protein substrate interaction of PE-H with PET, molecular docking with substrates MHET, BHET, and a previously reported PET tetramer (Joo et al., 2018) was conducted. Interestingly, wild type PE-H was not found to viably bind the tested substrates in the active site, but substrate molecules accumulated in an adjacent groove. For variant Y250S, viable binding modes for MHET and BHET, but not the PET tetramer were found. However, biochemical data revealed catalytic activity of PE-H and variant Y250S and PET film as a substrate. This observation might be connected to the flexibility of the active site of PE-H, suggesting that PE-H can rearrange its active site cleft to accommodate respective substrates. In line with that, flexibility of the active site was shown for PETase and reported to be crucial for the enzyme activity at room temperature (Fecker et al., 2018). Furthermore, MHET was accommodated in a catalytically viable position in PE-H Y250S; thus, the observed absence of MHET hydrolysis cannot be attributed to low affinity or preferred binding in a detrimental position for hydrolysis. Molecular reasons for that substrate preference might be addressed in further studies.

The two distinct binding poses observed for variant PE-H Y250S hint at a mechanism in which one polymer unit binds to the groove adjacent to the catalytic site, a second unit bridges the distance to the catalytic site, and a third unit is cleaved from the polymer chain. This mechanism with the adjacent groove acting as an anchor point for the polymer could increase the processivity of PE-H, similar to the adjacent substrate binding site in Hyal lyases (Rigden and Jedrzejas, 2003; Stern and Jedrzejas, 2008). A similar PET binding mechanism that involves a binding grove that stabilizes different units of a polymeric substrate was proposed for PETase binding elements of 2-HE(MHET)_4_ in subsites I, IIa, IIb, and IIc, facilitating the binding of the PET polymer (Joo et al., 2018). The presence of an adjacent substrate binding site may also contribute to the significant decrease in esterase activity of PE-H variants G254S, Y258N, and N259Q and the variant with the combined mutations. All three substitutions are located at the adjacent groove (Figure 7F) and alter their shape and molecular recognition properties, presumably hampering substrate binding that way.

As we did not obtain valid docking poses for 2-HE(MHET)_4_, we cannot comment on the *gauche*-to-*trans* ratio of the substrate’s OC–CO torsion angle when bound to the catalytic site, which has been discussed recently (Joo et al., 2018; Seo et al., 2019; Wei et al., 2019b). Our lowest-energy pose of 2-HE(MHET)_4_ (Figure 7B), which is located in the adjacent groove, shows all-*gauche* torsion angles. This result is in line with low *trans*-to-*gauche* ratios of (9 – 14): (91 – 86) experimentally determined for amorphous PET (Schmidt-Rohr et al., 1998; Wei et al., 2019b). We note, though, that even an increase in the *trans* content by a factor of ∼3 as discussed (Joo et al., 2018; Seo et al., 2019; Wei et al., 2019b) relates to changes in the conformational free energy of the substrate on the order of the thermal energy and, thus, may be compensated by favorable interactions with the protein.

## Conclusion

Polyester hydrolases are of considerable interest for a variety of biotechnological applications (Nikolaivits et al., 2018), for example the removal of cyclic PET oligomers from polyester fibers in the textile industry (Riegels et al., 1997). As far as biocatalytic degradation of PET in an industrial context is concerned, elevated temperatures close to the glass transition temperature of PET are desirable (Wei and Zimmermann, 2017). Hence, protein engineering of PE-H would be required to design a PE-H variant for efficient PET hydrolysis at elevated temperatures as has been demonstrated for PETase (Son et al., 2019). However, additional applications appear feasible for PE-H as it has been demonstrated that the PE-H homologous enzyme PpelaLip originating from the closely related genus *Pseudomonas pelagia* can be used for the biodegradation of different synthetic polyesters and may thus be applicable for waste water treatment (Haernvall et al., 2017). In this case, the activity of the respective biocatalyst at low temperatures is an advantage allowing for the hydrolysis of synthetic polymers at 15°C (Haernvall et al., 2018).

We have described here the identification, biochemical and structural characterization of the polyester hydrolase PE-H from the marine mesophilic bacterium *P. aestusnigri*. The crystal structure of PE-H represents the first structure of a type IIa PET hydrolytic enzyme thus closing the gap between PET hydrolytic enzymes of type I and IIb. We furthermore succeeded to significantly increase the enzymatic activity of PE-H toward different substrates by introducing a single amino acid substitution, namely Y250S. This substitution resulted in a rearrangement of the active site conformation, favored by prevention of a polar contact of Y250 located next to the catalytic active histidine, and a loop region of the active site cleft. Furthermore, a PET polymer binding mechanism was proposed based on molecular docking computations. Our results thus provide important information regarding structural features required for efficient polyester degradation and indicate that marine bacteria such as *P. aestusnigri* may prove as a prolific source for such enzymes.

## Data Availability Statement

Structures were deposited in the protein data bank (https://www.rcsb.org) under the accession codes 6SBN (WT PE-H) and 6SCD (PE-H Y250S).

## Author Contributions

K-EJ conceived the research concept. AB, ST, MF, and SS designed the experiments. AB, ST, and EK-G performed the experimental work. SK, AH, and SS performed the crystallization trials and structure solution. CG and HG conducted the molecular docking computations, discussed the results, and wrote the corresponding parts of the manuscript. AB, ST, and AH analyzed the data and wrote the draft manuscript. K-EJ and MF revised the manuscript. All authors read and approved the final manuscript.

## Conflict of Interest

HG and K-EJ are employed by Forschungszentrum Jülich GmbH. The remaining authors declare that the research was conducted in the absence of any commercial or financial relationships that could be construed as a potential conflict of interest.

## Footnotes

1 https://openwetware.org/wiki/Lidstrom:Autoinduction_Media

2 http://www.embl-hamburg.de/Auto-Rickshaw/

3 https://arpwarp.embl-hamburg.de

4 https://www.rcsb.org

5 https://pymol.org/2/

6 http://www.ebi.ac.uk/msd-srv/ssm/cgi-bin/ssmserver
