# Supplementary material for: A Novel Polyester Hydrolase From the Marine Bacterium Pseudomonas aestusnigri – Structural and Functional Insights
Source: Front Microbiol. 2020 Feb 13;11:114. doi: 10.3389/fmicb.2020.00114 (PMC7031157; doi:10.3389/fmicb.2020.00114)
Supplement: Supplementary file 1 [file Data_Sheet_1.PDF]

Supplementary Material

Type	Name	Alignment	Position
	LCC	---SNPYQRGPNPT	11
I	Cut190	--------------------------------------MRGSHHHHHHGSNPYERGPDPT	22
	TfCut2	---ANPYERGPNPT	11
IIb	PETase	--MNFPRASRLMQAAVLGGLMAVSA-------------------AATAQTNPYARGPNPT	39
	PET12	MPPDCVLPRRLAAAALLASATLVPL-------------------SAAAQTNPYQRGPDPT	41
IIa	PET5	--MN---KSILKKLSFGTSVLLVSMNALSWTPSPTPNPDPIPDPTPCQDDCDFTRGPNPT	55
	PE-H	MPFNK--KSVLA--LCGAGALLFSMSALANNPAP-------TDPGDSGGGSAYQRGPDPS	49
	LCC	RSALT-ADGPFSVATYTVSRLSVSGFGGGVIYYPTGTS-LTFGGIAMSPGYTADASSLAW	69
I	Cut190	EDSIEAIRGPFSVATERVSSF-ASGFGGGTIYYPRETDEGTFGAVAVAPGFTASQGSMSW	81
	TfCut2	DALLEARSGPFSVSEENVSRLSASGFGGGTIYYPREN--NTYGAVAISPGYTGTEASIAW	69
IIb	PETase	AASLEASAGPFTVRSFTVS-R-PSGYGAGTVYYPTNAG-GTVGAIAIVPGYTARQSSIKW	96
	PET12	TRDLEDSRGPFRYASTNVR-S-PSGYGAGTIYYPTDVS-GSVGAVAVVPGYLARQSSIRW	98
IIa	PET5	PSSLEASTGPYSVATRSVASS-VSGFGGGTLHYPTNTT-GTMGAIAVVPGFLLQESSIDF	113
	PE-H	VSFLEADRGQYSVRSSRVSSL-VSGFGGGTIYYPTGTT-GTMGAVVVIPGFVSAESSIDW	107
	LCC	LGRRLASHGFVVLVINTNSRFDYPDSRASQLSAALNYLRT---SSPSAVRARLDANRLAV	126
I	Cut190	YGERVASQGFIVFTIDTNTRLDQPGQRGRQLLAALDYLVE---RSDRKVRERLDPNRLAV	138
	TfCut2	LGERIASHGFVVITIDTITTLDQPDSRAEQLNAALNHMIN---RASSTVRSRIDSSRLAV	126
IIb	PETase	WGPRLASHGFVVITIDTNSTLDQPSSRSSQQMAALRQVASLNGTSSSPIYGKVDTARMGV	156
	PET12	WGPRLASHGFVVITLDTRSTSDQPASRSAQQMAALRQVVALSETRSSPIYGKVDPNRLAV	158
IIa	PET5	WGPKLASHGFVVITISANSGFDQPASRATQLGRALDYVINQSNGSNSPISGMVDTTRLGV	173
	PE-H	WGPKLASYGFVVMTIDTNTGFDQPPSRARQINNALDYLVSQNSRSSSPVRGMIDTNRLGV	167
	LCC	AGHSMGGGGTLRIAEQNPSLKAAVPLTPWHTDKT-FN-TSVPVLIVGAEADTVAPVSQHA	184
I	Cut190	MGHSMGGGGSLEATVMRPSLKASIPLTPWNLDKT-WGQVQVPTFIIGAELDTIAPVRTHA	197
	TfCut2	MGHSMGGGGSLRLASQRPDLKAAIPLTPWHLNKN-WSSVTVPTLIIGADLDTIAPVATHA	185
IIb	PETase	MGWSMGGGGSLISAANNPSLKAAAPQAPWDSSTN-FSSVTVPTLIFACENDSIAPVNSSA	215
	PET12	MGWSMGGGGTLISARDNPSLKAAVPFAPWHNTAN-FSGVQVPTLVIACENDTVAPISRHA	217
IIa	PET5	VGWSMGGGGALQLAS-GDRLSAAIPIAPWNQGGNRFDQIETPTLVIACENDVVASVNSHA	232
	PE-H	IGWSMGGGGTLRVAS-EGRIKAAIPLAPWDTTSYYASRSQAPTLIFACESDVIAPVLQHA	226
	LCC	IPFYQNLPSTTPKVYYELDNASHFAPN---SNNAAISVYTISWMKLWVDNDTRYRQFLCN	241
I	Cut190	KPFYESLPSSLPKAYMELDGATHFAPN---IPNTTIAKYVISWLKRFVDEDTRYSQFLCP	254
	TfCut2	KPFYNSLPSSISKAYLELDGATHFAPN---IPNKIIGKYSVAWLKRFVDNDTRYTQFLCP	242
IIb	PETase	LPIYDSMSR-NAKQFLEINGGSHSCANSGNSNQALIGKKGVAWMKRFMDNDTRYSTFACE	274
	PET12	SSFYNSFSSSLAKAYLEINNGSHTCANTGNSNQALIGKYGVAWIKRFVDNDTRYSPFLCG	277
IIa	PET5	SPFYNRIPSTTDKAYLEINGGSHFCANDGGSIGGLLGKYGVSWMKRFIDNDLRYDAFLCG	292
	PE-H	SPFYNSLPSSIDKAFVEINGGSHYCGNGGSIYNDVLSRFGVSWMKLHLDEDSRYKQFLCG	286
	LCC	VNDPALS---DFRTNNRHCQ----------------------	258
I	Cut190	NPTDR-----AIEEYRSTCPYKLN------------------	273
	TfCut2	GPRDGLF--GEVEEYRSTCPFYPNSSSVDKLAAALEHHHHHH	282
IIb	PETase	NPNSTRVSDFRTA----NCSLE-------------HHHHHH-	298
	PET12	APHQADLRSSRLSEYRESCPY---------------------	298
IIa	PET5	PDHAANR---SVSEYRDTCNY---------------------	310
	PE-H	PNHTSDS---QISDYRGNCPYL-------------EHHHHHH	312

Figure S1 Multiple sequence alignment of PE-H with amino acid sequences of different cutinases and PET hydrolases using the program Clustal Omega. The enzymes were assigned to different types of polyester hydrolases. Amino acid residues of the catalytic triad are marked by a red triangle, disulfide forming cysteine residues are highlighted in orange and connected by an orange line. Amino acids of the extended loop region, specific for type II PET hydrolases, are framed in red. Abbreviations are: leaf-branch compost metagenome cutinase (LCC); *Saccharomonospora viridis* cutinase (Cut190); *Thermobifida fusca* cutinase (TfCut2); *Ideonella sakaiensis* PET hydrolase (PETase); *Polyangium brachysporum* PET hydrolase (PET12); *Oleispira antarctica* PET hydrolase (PET5).

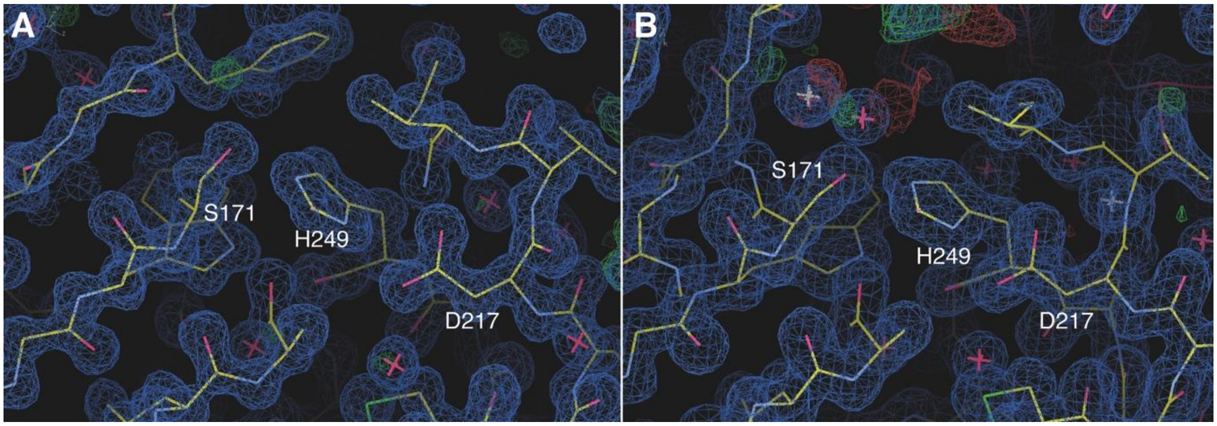

Figure S2 Electron densities around the active site of **(A)** PE-H WT (PDB code 6SBN) and **(B)** PE-H Y250S (PDB code 6SCD) with residues of the catalytic triad highlighted with labels. 2FoFc contoured at 1 σ.

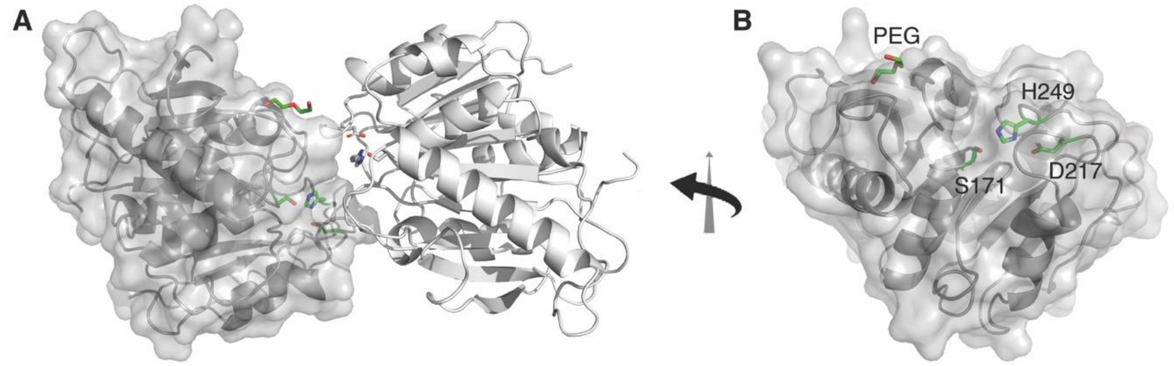

Figure S3 (**A**) Asymmetric unit containing two molecules of PE-H Y250S (PDB code 6SCD) depicted as cartoon showing the monomer with bound PEG molecule also in surface representation. In (**B**) the left molecule is rotated counter-clockwise with the residues of the catalytic triad as well as the PEG molecule shown as green sticks.

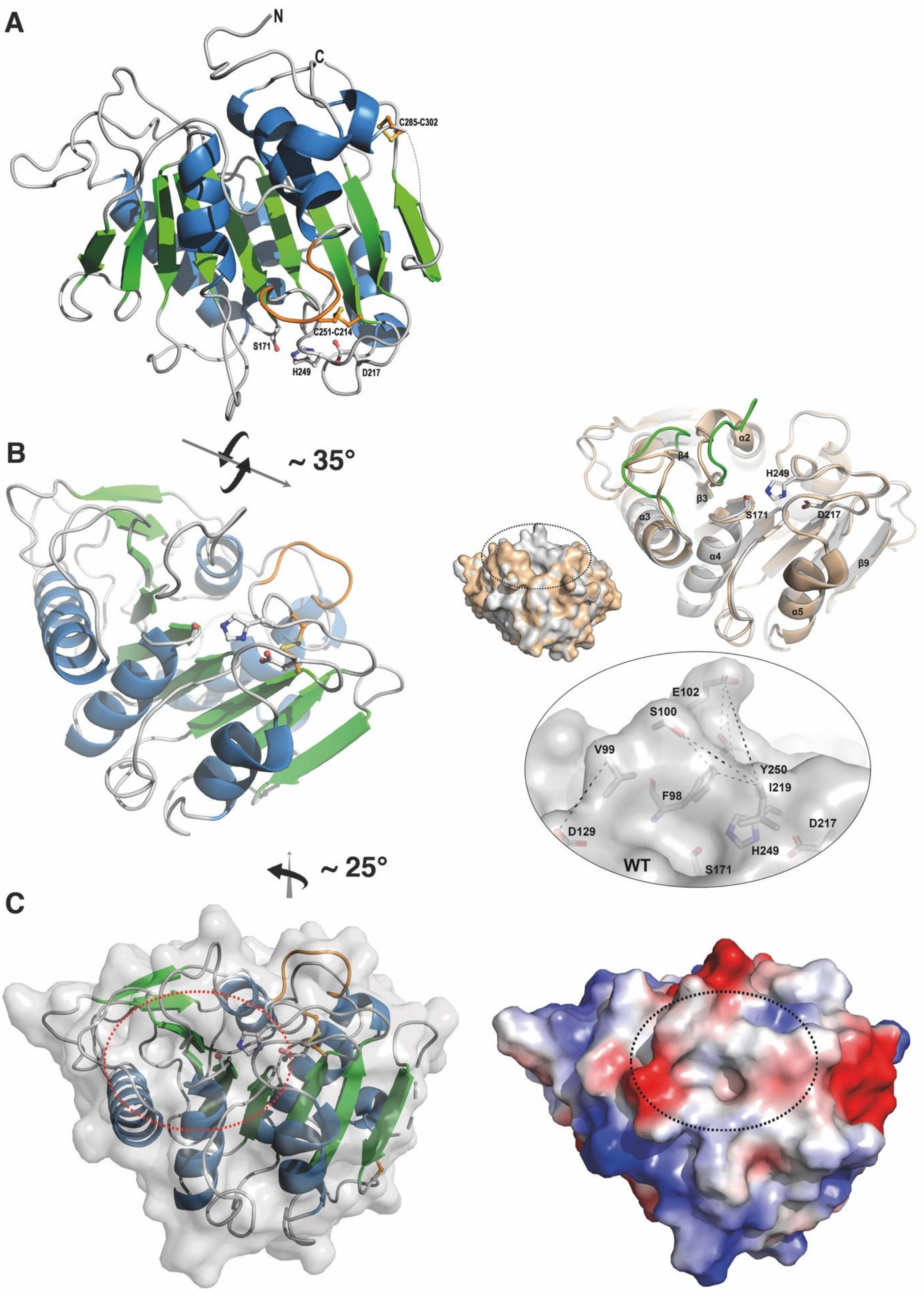

A
N
C
C285-C302
C251-C214
S171
H249
D217
B
~ 35°
α2
β4
H249
β3
S171
D217
α3
α4
β9
α5
E102
S100
V99
Y250
I219
F98
D217
D129
H249
WT
S171
~ 25°
C

Figure S4 (**A**) Orientation of PE-H as reference to Figure 4. (**B**) PE-H is rotated for around 35° from bottom to top to zoom in the active site (as in Figure 5). In (**C**) PE-H is additionally rotated counter-clockwise for around 25° for a better view on the active site cleft (as in Figure S5).

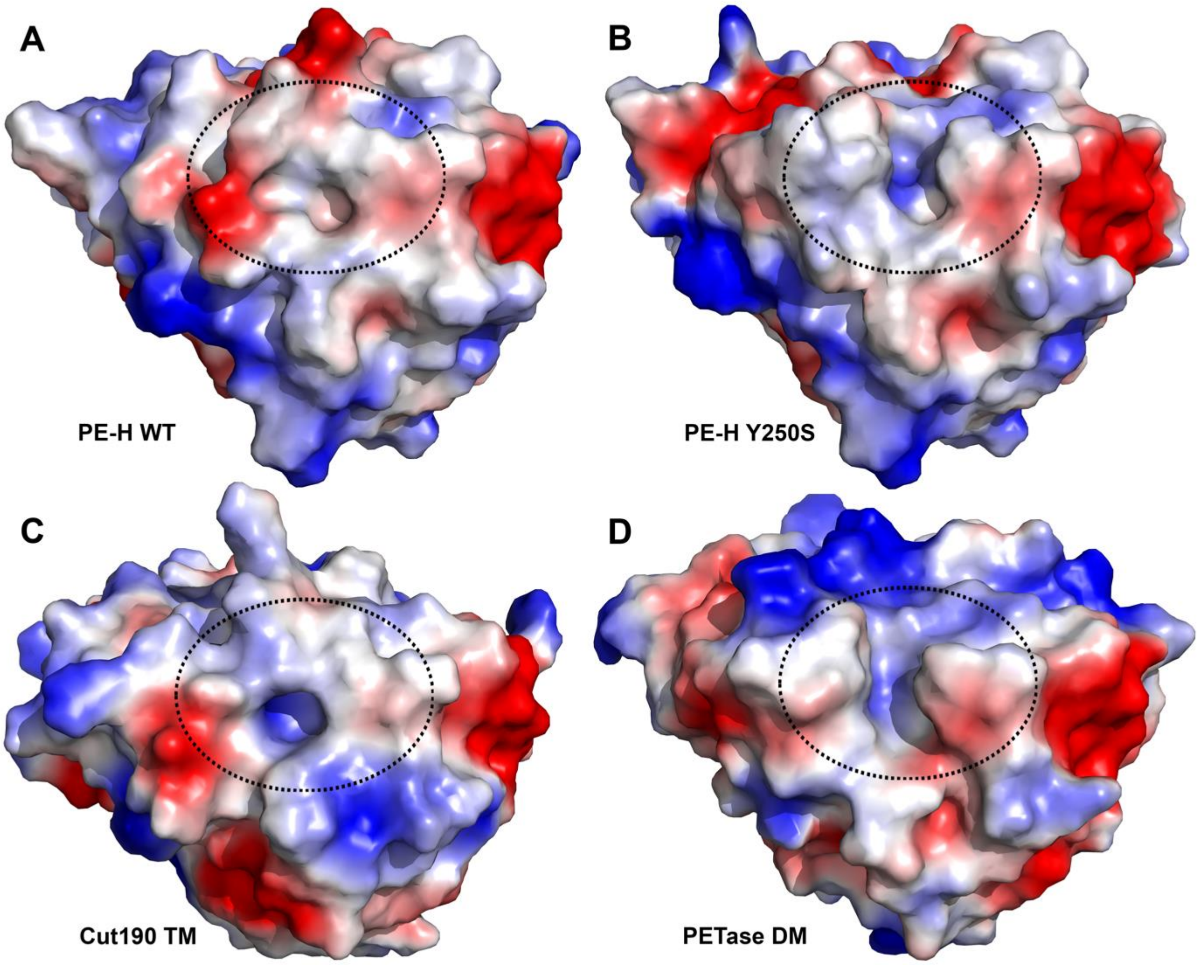

Figure S5 Surface representation of PE-H variants and two structural homologues. **(A)** shows wild type PE-H (PDB code 6SBN), **(B)** PE-H Y250S (PDB code 6SCD), **(C)** Cut190 triple mutant (TM) from *S. viridis* (PDB code 5ZRR) and **(D)** PETase double mutant (DM) from *I. sakaiensis* (PDB code 5XH3). Color code of the electrostatic surface is blue for positive, red for negative charge. All molecules are shown in identical orientation with the active site cleft positioned in the middle and marked by a dashed line.

Table S1 Amino acid composition of PETase (6EQE) and PE-H (6SBN) at selected positions as marked in the main text (i to iii).

	active site			(i)	(ii)	(iii)					
PETase	S160	D206	H237	S238	W159	S242	G243	N244	S245	N246	Q247
PE-H	S171	D217	H249	Y250	W170	G254	G255	S256	I257	Y258	N259

Table S2 Thermal melting points (Tm) of PE-H (WT) and different variants determined by nano differential scanning fluorimetry. The top row shows the respective single amino acid substitutions. Mutations G254S, S256N, I257S, Y258N, and N259Q were combined (ext.) to resemble the amino acid combination of the extended loop region of PETase.

	WT	G254S	S256N	I257S	Y258N	N259Q	ext.	Y250S
Tm [°C]	50.8	39.7	49.5	48.5	43.8	45.2	39.4	49.8

Table S3 Data collection and refinement statistics of PE-H

	**PE-H**	**PE-H Y250S**
**Beamline**	ID29, ESRF, Grenoble	P13, DESY, Hamburg
**Crystal parameters**		
Space group	C 2 2 21	I 21 21 21
Unit cell parameters:<br>a, b, c (Å)<br>α, β, ɣ (°)	 68.81, 80.01, 88.91<br>90, 90, 90	 94.53, 98.27, 121.23<br>90, 90, 90
**Data collection and processing**		
Wavelength	0.9762	0.9505
Resolution (Å)	50.00 – 1.09 (1.15 – 1.09)	77.34 – 1.35 (1.42-1.35)
Total reflections	1293506 (198125)	494296 (78069)
Unique reflections	101774 (16134)	122029 (19634)
Multiplicity	12.7 (12.3)	4.1 (4.0)
Completeness (%)	99.5 (98.4)	99.11 (99.26)
I/σ(I)	16.20 (4.70)	14.98 (2.52)
Wilson B-factor ($Å^2$)	10.4	16.7
R-merge	0.076 (0.429)	0.043 (0.439)
R-meas	0.080 (0.448)	0.050 (0.505)
CC 1/2	0.999 (0.970)	0.998 (0.849)
**Refinement statistics**		
Reflections used in refinement	99673	116011
Reflections used for R-free	2057	5715
R work (%)	10.69	11.3
R free (%)	13.70	15.2
Number of non-hydrogen atoms<br>macromolecules<br>ligands<br>solvent	2401<br>2028<br>5<br>368	4590<br>4048<br>49<br>493
RMS (bonds)<br>RMS (angles)	0.027<br>2.206	0.026<br>2.430
Ramachandran plot:<br>favoured (%)<br>Allowed (%)<br>outliers (%)	 96.5<br>2.7<br>0.8	 97.3<br>2.5<br>0.2
Average B-factor ($Å^2$)	14.0	22.0
**Model content**		
Monomers ASU	1	2
Protein residues	263 (38-285; 292-306)	532 (40-305)
Ligand	1 ACT, 1 NA	1 SO4, 3 PO4, 1 ACT, 1 PEG, 2 GOL, 3 CL, 3 NA
Waters	368	495
**PDB code**	6SBN	6SCD

(Statistics for the highest resolution shell are shown in parentheses)

Table S4: Structure based alignment of wild type PE-H and PE-H Y250S against pdb entries. Top 10 of the most similar structures are given with their corresponding alignment quality (Q-score), root mean square deviation (RMSD), number of aligned residues (N_{algn}), their sequence identity (Seq-%), their PDB number (Target PDB), and description of the PDB entry (Protein).

**Wild type PE-H** (pdb-code: 6SBN)						
**##**	**Q-score**	**RMSD**	**N_{algn}**	**Seq-%**	**Target PDB**	**Protein**
**1**	0.76	1.18	246	51	5zrr:A	**cutinase** Cut190 S176A/S226P/R228S mutant (*Saccharomonospora viridis*)
**2**	0.75	1.20	245	51	5xg0:B	*1* **PET hydrolase** *(Ideonella sakaiensis)*
**3**	0.75	1.18	244	51	6ane:A	*2* **PET hydrolase** *(Ideonella sakaiensis)*
**4**	0.75	1.24	245	51	4cg2:A	***3*** **PET hydrolase** *(Thermobifida fusca)*
**5**	0.75	1.25	245	51	4cg1:A	***4*** **PET hydrolase** *(Thermobifida fusca)*
**6**	0.74	1.22	240	53	5luj:A	***5*** **cutinase 2** *(Thermobifida cellulosilytica)*
**7**	0.74	1.25	244	52	5lul:A	*6* **cutinase 2** R19S/R29N/A30V mutant *(Thermobifida cellulosilytica)*
**8**	0.74	1.32	246	52	5luk:A	*7* **cutinase 2** R29N/A30V mutant *(Thermobifida cellulosilytica)*
**9**	0.73	1.28	245	51	5lui:A	**cutinase 1** (*Thermobifida cellulosilytica*)
**10**	0.73	1.25	242	52	5zoa:A	***8*** **cutinase** *(Thermobifida fusca)*
**PE-H Y_{250}S** chain A (pdb-code 6SCD)						
**1**	0.84	1.17	259	51	5xh3:A	**9** **PET hydrolase** R103G/S131A mutant (HEMT complex) *(Ideonella sakaiensis)*
**2**	0.84	1.17	259	51	5xfz:A	**10** **PET hydrolase** R103G/S131A mutant *(Ideonella sakaiensis)*
**3**	0.83	1.11	257	51	6qgc:A	**11** **PETase** *(Ideonella sakaiensis)*
**4**	0.83	1.14	257	51	5xfy:A	**12** **PET hydrolase** S131A mutant *(Ideonella sakaiensis)*
**5**	0.83	1.16	257	51	5xh2:A	**13** **PET hydrolase** R103G/S131A mutant (pNP complex) *(Ideonella sakaiensis)*
**6**	0.83	1.10	256	52	6eqg:C	**14** **PET hydrolase** *(Ideonella sakaiensis)*
**7**	0.83	1.08	253	52	4wfk:A	**15** **cutinase** Cut190 S226P mutant (Ca(2+)-bound state) *(Saccharomonospora viridis)*
**8**	0.83	1.08	253	52	4wfj:A	**16** **cutinase** Cut190 S226P mutant (Ca(2+)-bound state) *(Saccharomonospora viridis)*
**9**	0.83	1.08	255	52	6eqg:B	**17** **PET hydrolase** *(Ideonella sakaiensis)*
**10**	0.83	1.07	255	52	6eqh:A	**18** **PET hydrolase** *(Ideonella sakaiensis)*
